# The impact of COVID-19 pandemic on hand hygiene compliance of healthcare workers in a tertiary hospital in East China

**DOI:** 10.3389/fmed.2023.1160828

**Published:** 2023-06-22

**Authors:** Xing Zhang, Yonghui Ma, Li Kong, Yusen Li, Juan Wang, Ning Li, Yujie Xia, Peng Wang, Min Zhang, Lili Liu, Dingding Zhang, Luhan Wen, Shuangshuang Wang, Zhenghui Liu, Xia Yue, Jixia Wang, Tong Zhang, Xiujuan Meng

**Affiliations:** Healthcare-Associated Infection Control Department, Affiliated Hospital of Jining Medical University, Jining, Shandong, China

**Keywords:** healthcare workers, hand hygiene performance, COVID-19, prevention and control strategies, hand hygiene, coronavirus disease 2019

## Abstract

**Introduction:**

Hand hygiene is a cost-effective measure to reduce healthcare-associated infections (HAIs) in healthcare facilities. The impact of the coronavirus disease 2019 (COVID-19) pandemic on hand hygiene performance (HHP) provided evidence for targeted hand hygiene intervention measures.

**Methods:**

This study evaluated the HHP rate in a tertiary hospital before and after the COVID-19 outbreak. HHP was checked by infection control doctors or nurses every day, and they inputted the HHP rate to the full-time infection control staff every week. A random examination of HHP was conducted by a confidential worker every month. The HHP of healthcare workers (HCWs) was monitored in the outpatient department, inpatient ward, and operating room from January 2017 to October 2022. The influence of COVID-19 prevention and control strategies on HHP was elucidated by analyzing the results of HHP during the study period.

**Results:**

The average HHP rate of HCWs was 86.11% from January 2017 to October 2022. The HHP rate of HCWs after the COVID-19 pandemic was statistically significantly higher than that before the pandemic (*P* < 0.001). The HHP rate was the highest (93.01%) in September 2022 when the local epidemic occurred. Among the different occupation categories, medical technicians showed the highest HHP rate (89.10%). The HHP rate was the highest after contact with body fluids or blood of patients (94.47%).

**Conclusion:**

The HHP rate of HCWs in our hospital showed an increasing trend in the recent 6 years, especially during the COVID-19 pandemic, and the increase was most obvious during the local epidemic.

## Introduction

Healthcare-associated infections (HAIs) are the most prevalent adverse events of hospital care, posing a substantial threat to patient safety, which have an association with increased financial burden and mortality (1, 2). HAIs are mainly transmitted through the contaminated hands of healthcare workers (HCWs). Therefore, intervention strategies to keep hands clean are particularly important. According to the specifications of hand hygiene for healthcare workers in China, hand hygiene refers to the general term of handwashing, hygienic hand disinfection, and surgical hand disinfection used by healthcare workers during their professional activities. It has been well-established that hand hygiene (HH) is a cost-effective measure to reduce HAIs (3). A new report from the World Health Organization (WHO) (4) showed that 70% of HAIs can be prevented by following good HH and other cost-effective practices. Meanwhile, another survey (5) reported that a 10% improvement in hand hygiene can reduce the overall incidence of HAIs by 6%. A 6-year study (6) found the number of HAIs decreased from 2,012 to 1,831, and their incidence per 1,000 patient-days fell from 14.0 to 11.7 (*P* < 0.0001) during the study period, and there was a weak but statistically significant negative correlation between the monthly incidence of HAIs and HH compliance. Therefore, hand hygiene is the simplest, the most effective, the most convenient, and the most economical means of infection control.

However, the present situation of HH of HCWs at home and abroad is not satisfactory. Erasmus and Tjbrug (7) conducted a systematic review of research papers on hand hygiene compliance published before 2009 and found that HHP rate was 40% on average; the HHP rate in an intensive care unit (30–40%), which was lower than that in other departments (50–60%); and the HHP rate of doctors (32%) was lower than that of nurses (48%). A 3-year (2017–2019) study by Belkebir et al. (8) found HHP rates of nurses in 3 years were 36, 59, and 64%, respectively, while HHP rates of doctors were 42, 56, and 58%, respectively. In 2015, Xu et al. (9) conducted a retrospective survey of HHP in more than 200 hospitals across the country by means of multi-center research, and the results showed that the average HHP in hospitals was 70.10%, and the average accuracy of HH was 71.90%.

In a laboratory experiment, SARS-CoV-2 was found to be stable on the skin for up to 14 days at 4°C, 96 h at 22°C, and for at least 8 h at 37°C (10). With the global spread of COVID-19 in 2020, HH is becoming an important measure to prevent and control the spread of COVID-19 (11, 12). Although HH might seem simple, the HHP rates were different as reported by some studies and were obviously low in some areas (13–15). To study the HHP of HCWs in our hospital in the recent 6 years, especially the impact of prevention and control strategies on HHP during the COVID-19 epidemic, we analyzed the HHP of HCWs from January 2017 to October 2022, evaluated the effect of prevention and control strategies on HHP, and proposed some suggestions for improving HHP in the future.

## Materials and methods

### Hospital setting and study subjects

This study was carried out in a university teaching hospital in Jining, Shandong Province from January 2017 to October 2022. A new hospital area was added in 2021. Therefore, this hospital currently has 4,100 beds and 5,254 healthcare workers. With the updating and development of disciplines, the hospital currently has 82 clinical departments and 15 intensive care units, responsible for the healthcare of over 20 million people in the southwestern region of Shandong Province, China. In 2021, the hospital had 4 million outpatients, 199,000 discharged patients, 128,000 operations, and 6.6 days of hospitalization on average. This study analyzed the HHP of HCWs in the outpatient department, inpatient ward, and operating room. The study subjects were related personnel engaged in daily diagnosis and treatment, cleaning, and logistics activities in the hospital, including medical personnel, nursing personnel, medical technical personnel, and workers (cleaning and logistics support), including training personnel, standardized training personnel, and interns.

### Ethics approval and consent to participate

This study was approved by the Ethics Committee of the Affiliated Hospital of Jining Medical University (2022-12-C018). The application for exemption of informed consent has been approved. The reasons for the exemption of informed consent are as follows: First, the Affiliated Hospital of Jining Medical College formulated and issued the HH management system (M-9900101-006) and implemented it. HCWs were informed through the system that they would be monitored for HH. Second, this study was based on the monitoring method of HHP in Appendix D of the Health Industry Standard of the People's Republic of China for Medical Personnel Hand Hygiene (WS/T-2019). According to our method, the direct observation method was adopted. The observation objects were randomly selected, and the timing and implementation of HH of HCWs were observed and recorded without informing them. If informed consent was obtained, it could not reflect the real situation of HH of HCWs. Finally, this study was retrospective. We collected the data of the study object, and the privacy of all participants was anonymous in our study.

### Study contents and methods

We issued a recruitment notice for HCWs to recruit 20 dispatched nurses as confidential observers for hand hygiene. First of all, we provided unified training for confidential observers, including the purpose, plan, content, and method of the investigation. Direct observers and confidential observers should master unified standards, use unified questionnaires, and ensure the accuracy of observation records. Second, an on-site examination would be conducted after the training, only those who passed the examination were allowed to take up their positions. We provided a certain economic subsidy to the confidential observers every year.

**Figure 1:**
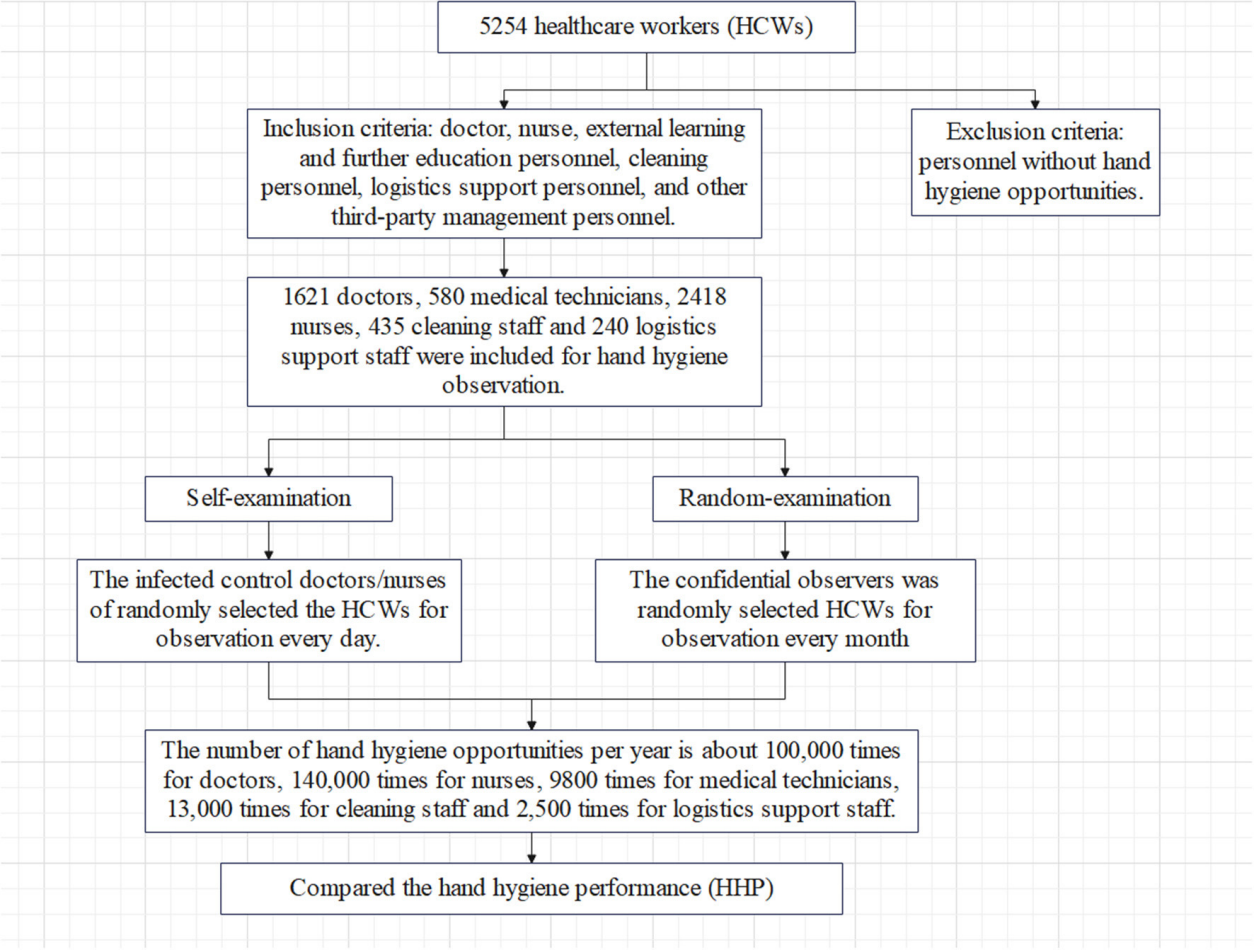
Research technology roadmap.

We screened individuals who meet the inclusion criteria and exclusion criteria for observation. Inclusion criteria: healthcare workers with observation opportunities for hand hygiene, including doctors, nurses, external learning and further education personnel, cleaning personnel, logistics support personnel, and other third-party management personnel. Exclusion criteria: patient caregivers and personnel without hand hygiene opportunities. The investigation process is detailed in Figure 1.

HHP was measured during healthcare work by direct observation by infection control doctors or nurses every day (self-examination), and they inputted the HHP results to the full-time infection control staff every week. A random examination of HHP was conducted anonymously by the confidential worker every month. Direct observation method or viewing surveillance video method was adopted by the confidential worker. Without informing the subjects, the confidential worker randomly selected the subjects and randomly observed them in daily professional activities. It was ensured that one healthcare worker was not observed for more than three hand hygiene times at a time and the duration of observation did not exceed 20 min. No <80 hand hygiene sessions per occupation per month were observed.

One confidential worker was responsible for several departments. To avoid discovering the identity of the confidential worker, the departments investigated by the confidential worker were adjusted every month. Two groups of observers covered the clinical departments of the entire hospital every month. The observation parameters included the observation date, observation start and end time, observation place, observation personnel, occupation category of observed personnel (doctors, nurses, medical technicians, cleaning staff, etc.), hand hygiene indicators, whether performing hand hygiene and hand hygiene methods (handwashing, hygienic hand disinfection, and surgical hand disinfection), and the accuracy of hand hygiene methods. Monthly reports of hand hygiene observation were obtained through the infection surveillance information system.

### Training and quality control

Training on HHP monitoring was conducted every year for infection control doctors or nurses and confidential workers, including hand hygiene observation requirements, hand hygiene indications, and the use of the infection monitoring information system. Hand hygiene observers were retrained if changed. The full-time infection control staff of the hospital analyzed the feedback on the monitoring situation of HHP of the entire hospital every quarter.

According to the WHO guidelines on hand hygiene in healthcare (16), the five opportunities of hand hygiene include before contact with the patient, before performing cleaning or aseptic procedures (including invasive procedures), after contact with the patient, after exposure to the body fluids of the patient (including contact with the patient's mucous membranes, broken skin or wounds, blood, body fluids, secretions, excreta, wound dressings, etc.), and after contact with the patient's surroundings (including contact with the patient's medical-related instruments, utensils, and other surfaces).

### Prevention and control strategy for the COVID-19 outbreak

**Figure 2:**
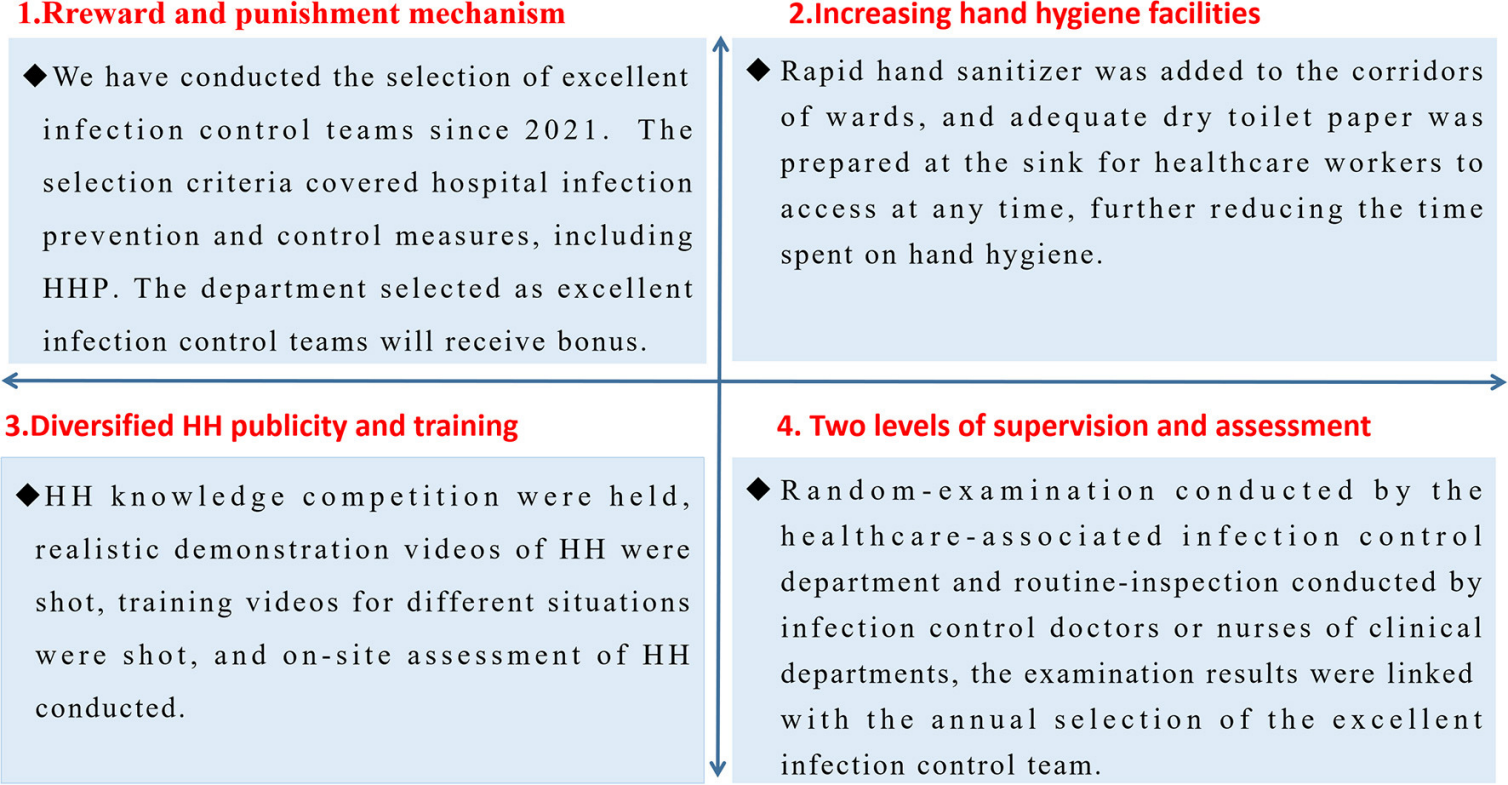
Hand hygiene compliance improvement strategy.

After the COVID-19 outbreak, to improve HHP, hand hygiene management measures were actively adopted to strengthen the hand hygiene management of healthcare workers. The detailed methods are shown in Figure 2.

### Data analysis

All the data were analyzed using SPSS 20.0 software (IBM Corp., Armonk, NY, USA). HHP rate = hand hygiene execution time number/hand hygiene execution time number × 100%. We compared the HHP rates of different survey times and methods (self-examination, random examination) and also compared the HHP rates of five hand hygiene opportunities and different professions of HCWs during different survey times. All comparisons were statistically assessed using Pearson's chi-square test, with levels of significance being set at 5% (*p* < 0.05, two-tailed).

## Results

### HHP rate of healthcare workers

**Figure 3:**
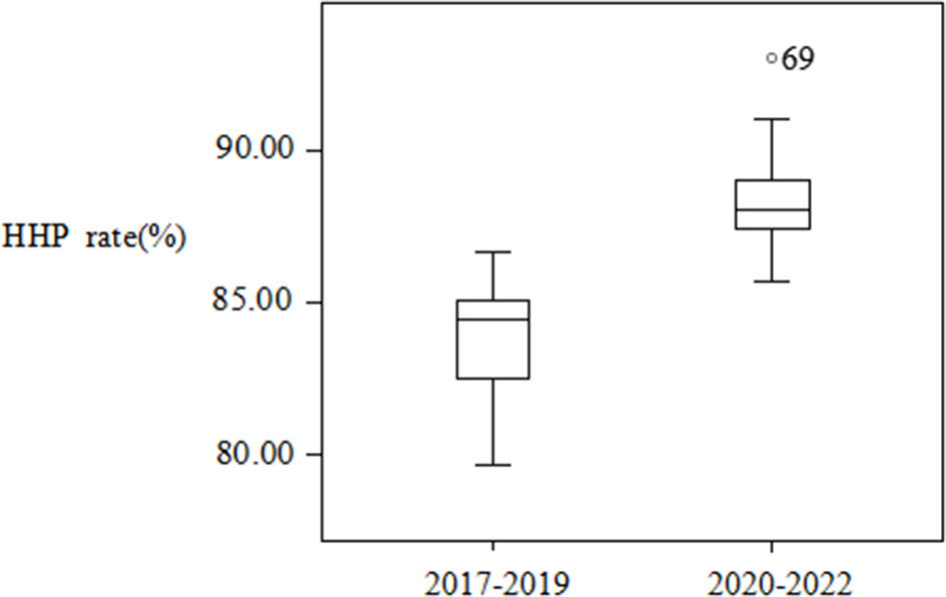
Distribution of the HHP rate of HCWs before and after the COVID-19 epidemic.

Data from 2017 to 2022 were collected, and they contained 1,627,728 hand hygiene opportunities in total. The average HHP rate of HCWs was 86.11% from January 2017 to October 2022. The HHP rate was the lowest in 2017 (81.99%) and the highest in 2022 (January–October; 89.50%). The HHP rate checked by infection control doctors or nurses was significantly higher than that checked by confidential workers (χ^2^ = 22,358.76, *P* < 0.001). The HHP rates showed an increasing trend every year in the recent 6 years, and the difference was statistically significant (χ^2^ = 8,481.636, *P* < 0.001). The average HHP rates of HCWs were 83.77% from 2017 to 2019 and 88.25% from 2020 to 2022. The difference in the HHP rate before and after the COVID-19 epidemic was statistically significant (χ^2^ = 6,818.435, *P* < 0.001; Table 1). The 69 index in Figure 3 is based on the outlier value corresponding to row 69, which was 93.01% of the HHP rate in September 2022.

**Table 1:** The HHP rate of HCWs from January 2017 to October 2022.

**Time**	**Self-examination**		**Random examination**		**Summation**	
	**Number of HHP (times)**	**HHP rate (%)**	**Number of HHP (times)**	**HHP rate (%)**	**Number of HHP (times)**	**HHP rate (%)**
2017	227,925	84.64	38,798	66.44	266,723	81.99
2018	239,556	85.91	22,640	71.33	262,196	84.65
2019	227,178	86.12	22,469	70.71	249,647	84.73
2020	243,846	88.23	18,303	73.87	262,149	87.22
2021	283,649	88.93	34,103	80.58	317,752	88.04
2022 (January to October)	248,105	90.17	21,156	81.63	269,261	89.50
Total	1,470,259	87.43	157,469	73.72	1,627,728	86.11

### HHP rate of medical personnel in different occupations

The average HHP rate was the highest among HCWs (89.10%), followed by nurses (87.37%), doctors (86.85%), workers (85.86%), doctors undergoing further study/internship/regular training (79.48%), and nurses in training/practice (77.97%). The lowest HHP rate was observed among the cleaning staff (72.11%). The HHP rates showed a gradually increasing trend in the recent 6 years, and the difference was statistically significant (χ^2^ = 14,612.087, *P* < 0.001). The HHP rates of doctors, nurses, medical technicians, refresher/intern nurses, cleaning staff, and service workers showed an increasing trend in the recent 6 years (*P* < 0.001; Table 2).

**Table 2:** HHP rate of hand hygiene of HCWs in different occupations.

**Occupation**	**2017**		**2018**		**2019**		**2020**		**2021**		**2022**		**χ^2^**	***P***
	**Number of HHP (times)**	**HHP rate (%)**	**Number of HHP (times)**	**HHP rate (%)**	**Number of HHP (times)**	**HHP rate (%)**	**Number of HHP (times)**	**HHP rate (%)**	**Number of HHP (times)**	**HHP rate (%)**	**Number of HHP (times)**	**HHP rate (%)**		
Doctor	95,885	82.52	94,024	85.44	90,238	85.20	95,540	87.73	118,095	88.89	105,845	90.35	3,581.773	<0.001
Nurse	137,363	84.26	131,923	86.30	123,331	86.59	129,841	88.35	152,419	88.91	130,512	89.68	2,483.379	<0.001
Medical technicians	7,091	87.36	8,946	86.77	9,174	88.53	10,809	89.45	13,532	90.67	9,825	90.42	128.802	<0.001
Refresher/intern nurse	6,676	73.28	6,994	77.42	6,829	78.25	4,532	80.19	4,267	80.81	3,175	81.48	142.843	<0.001
Further study/ internship/ training doctor	5,301	75.08	5,269	78.76	4,380	78.81	3,663	82.28	8,272	80.39	6,089	81.49	102.736	<0.001
Cleaners	12,811	58.63	12,676	66.63	12,880	66.90	15,052	76.06	17,981	77.97	11,313	84.89	2,864.138	<0.001
Service worker	1,596	78.20	2,364	84.69	2,815	82.66	2,712	86.98	3,186	89.67	2,502	89.37	169.922	<0.001

### HHP rate of HCWs under different hand hygiene opportunities

The average HHP rate of HCWs was the highest (94.47%) after contact with body fluids or blood of patients and the lowest (77.79%) after contact with patients' surroundings. The HHP rate of HCWs at different hand hygiene opportunities had statistical differences (χ^2^ = 35,276.383, *P* < 0.001). The HHP rate of different hand hygiene opportunities showed an increasing trend every year in the recent 6 years, with the differences being statistically significant (*P* < 0.001; Table 3).

**Table 3:** HHP rate of HCWs under different hand hygiene opportunities.

**Hand hygiene opportunity**	**2017**		**2018**		**2019**		**2020**		**2021**		**2022**		**χ^2^**	***P***
	**Number of HHP (times)**	**HHP rate (%)**	**Number of HHP (times)**	**HHP rate (%)**	**Number of HHP (times)**	**HHP rate (%)**	**Number of HHP (times)**	**HHP rate (%)**	**Number of HHP (times)**	**HHP rate (%)**	**Number of HHP (times)**	**HHP rate (%)**		
After contact with patients	68,844	83.32	67,048	86.05	63,676	85.73	65,749	88.28	79,653	88.53	66,999	89.84	1,705.242	<0.001
Before contacting patients	77,969	82.12	74,154	84.67	69,292	85.57	71,449	87.40	87,147	88.3	71,673	89.89	2,502.142	<0.001
After contact with patient's surroundings	56,699	71.16	54,681	74.97	55,085	75.36	60,725	79.39	72,544	80.93	58,735	83.54	3,511.539	<0.001
After contact with patient's body fluids or blood	27,063	92.44	28,821	94.08	27,722	93.83	28,083	95.74	33,937	95.25	30,385	95.21	403.443	<0.001
Before aseptic or cleaning operations	36,148	88.37	37,492	89.00	33,872	88.94	36,143	91.49	44,471	92.71	41,469	92.54	886.511	<0.001

### HHP rate of HCWs from January 2022 to October 2022

**Figure 4:**
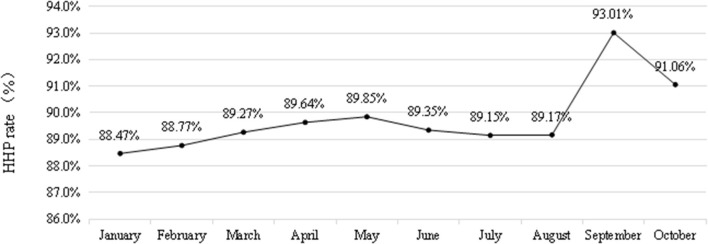
The HHP rate of HCWs from January 2022 to October 2022.

The average HHP rate of HCWs from January to October 2022 was 89.50% (240,989/269,261), among which the month of September showed the highest HHP rate of 93.01% (8,716/9,371). The HHP rate was the lowest (88.47%) in January (26,260/29,684). A comparison of the HHP rate of healthcare workers from January to October 2022 revealed a statistically significant difference (χ^2^ = 248.286, *P* < 0.001; Figure 4).

## Discussion

Hand hygiene is a critical component for preventing the transmission of pathogens causing HAIs. In this study, we analyzed the results of the HHP of HCWs in a tertiary hospital in recent 6 years. Moreover, we studied the impact of the COVID-19 pandemic on hand hygiene compliance of HCWs in our hospital. We compared the HHP rate of HCWs at different survey times using different survey methods (self-examination and random examination) in different hand hygiene opportunities and different occupations. It was shown that hand hygiene compliance was improved after the COVID-19 pandemic in our study.

The average HHP rate of HCWs in the recent 6 years was 86.11%, and HHP rates increased from 81.99% in 2017 to 89.50% in 2022 in this study; the difference in the HHP rate in the recent 6 years was statistically significant. There was a downward trend in hand hygiene compliance in 2019 compared with that in 2018. However, an obvious upward trend was observed after the COVID-19 outbreak in 2020. The difference in the HHP rate before and after the COVID-19 outbreak was statistically significant. During the epidemic period, the HAI management departments improved their prevention and control strategies, and simultaneously, the hand hygiene awareness of HCWs was strengthened. Therefore, the HHP rate significantly increased after the COVID-19 outbreak. This is consistent with the results reported by Liu et al. (17). The HHP rates of HCWs in hospitals increased annually from 2016 to 2020 in this study. Other studies also showed that the hand hygiene compliance of HCWs improved during the COVID-19 pandemic (18, 19).

Self-examination and random examination were used to monitor HHP in this study, and the HHP rates were calculated as 87.43 and 73.72%, respectively; the HHP rate was significantly lower in the random examination than that in the self-examination. The choice of monitoring methods is important, and we found that the hand hygiene examination method had a great impact on the HHP. This is consistent with the results of a study on the hidden hand hygiene investigation and analysis of eight comprehensive hospitals in Beijing (20). However, in both examination methods, the HHP rate showed an increasing trend, indicating that the healthcare workers paid more attention to hand hygiene, which to some extent reflects the effectiveness of HAI management in this hospital in recent years.

In this study, the HHP rate of HCWs in different occupations was different as well as the highest. The HHP rates of doctors undergoing further study/internship/regular training, nurses in training/practice, and cleaning staff were low, a finding similar to that was reported by other studies (21, 22). The cleaning staff had low knowledge level and poor self-protection awareness, which were likely to result in low rates in another study as well (23). We propose hand hygiene training for different occupation categories in the future. In particular, personnel going for further study, internship, and regular training should undergo hand hygiene training in conjunction with medical education (24). Regular assessments for different personnel should be conducted, and personalized training should be strengthened for different personnel. Hand hygiene pictures should be pasted in the cleaning room, and hand hygiene education videos should be made. Hand creams, hand washes, and other products should be awarded to those with high hand hygiene compliance.

The HHP rate of HCWs was the highest after contact with body fluids or blood of patients; meanwhile, it was the lowest after contact with patients' surrounding environment in our study, which was consistent with other studies (25–27). This indicated that HCWs focused on hand hygiene after performing the contamination operation and had a strong awareness of self-protection. However, HCWs mistakenly believed that they had no contact with patients but just contacted the surrounding environment without the possibility of contamination, thus ignoring HH after contact with the patient's surrounding environment (28). HCWs should realize that the purpose of hand hygiene is two-way protection, which can prevent the transmission of pathogens from patients to HCWs, as same as prevent the transmission of pathogens from HCWs to patients (29, 30).

Hand hygiene has never been more important than in the COVID-19 pandemic (31). The results of the present study demonstrated that the HHP rate of HCWs in the first 3 years (2017–2019) was lower (83.77%) than that in the latter 3 years (2020–2022) after the COVID-19 epidemic (88.25%), with the difference being statistically significant. This finding was similar to that reported by Zhao et al. (32) during the COVID-19 epidemic. The HHP rate of HCWs was highest in September 2022, which was due to the local epidemic in the hospital. There were several reasons for this result. First, because of the local COVID-19 epidemic, most people were isolated at home, and the number of inpatients was less than usual; therefore, they had sufficient time to perform hand hygiene. Second, hand hygiene awareness of HCWs improved, which promoted the implementation of hand hygiene (33).

In this study, there were several limitations. First, the direct observation method was used in the monitoring of HHP, which may be influenced by the Hawthorne effect. Second, this study had not been combined with the consumption of hand hygiene products to evaluate the HHP of HCWs. In future studies, the direct observation method can be combined with the consumption of hand hygiene products to evaluate the hand hygiene compliance of HCWs in a more objective manner.

## Conclusion

In brief, due to the COVID-19 epidemic, we developed a relatively perfect hand hygiene monitoring system, strengthened hand hygiene training, and increased the corresponding reward and punishment system; therefore, the HHP rate improved. Improving hand hygiene compliance is a continuous improvement effort, and we will develop some improvement strategies in the future based on the current survey results. A long-term hand hygiene mechanism should be established to maintain the best effect of hand hygiene compliance.

## Data availability statement

The original contributions presented in the study are included in the article, further inquiries can be directed to the corresponding author.

## Ethics statement

This study was approved by the Ethics Committee of Affiliated Hospital of Jining Medical University, China. Written informed consent from the participants was not required to participate in this study in accordance with the national legislation and the institutional requirements.

## Author contributions

PW, YM, LW, YX, NL, DZ, TZ, and LL assisted in data collection. JiW, YM, XY, JuW, and MZ conducted the experiments. XZ, SW, and TZ analyzed the results. XM and LK conceived the study. XZ and XM supervised the study and prepared the manuscript. All authors read and approved the final manuscript.
